# Effects of Curcumin on Inflammation in a Rat Model of Diabetic Retinopathy

**DOI:** 10.3390/life16091427

**Published:** 2026-08-27

**Authors:** Hyo Seon Yu, Ji Hye Kang, Seo Hyun Kim, Heeyoon Cho, Seong-Ho Koh, Eun Hee Hong, Yong Un Shin

**Affiliations:** 1 Graduate School of Biomedical Science and Engineering, Hanyang University, #222-1 Wangsimni-ro, Seongdong-gu, Seoul 04763, Republic of Korea; hello4144@hanyang.ac.kr (H.S.Y.); kangjh7380@naver.com (J.H.K.); joyksh010211@gmail.com (S.H.K.); 2 Department of Ophthalmology, Hanyang University College of Medicine, #222-1 Wangsimni-ro, Seongdong-gu, Seoul 04763, Republic of Korea; hycho@hanyang.ac.kr; 3 NOON Eye Clinic, Guri 11926, Republic of Korea; 4 Department of Neurology, Hanyang University College of Medicine, #222-1 Wangsimni-ro, Seongdong-gu, Seoul 04763, Republic of Korea; ksh213@hanyang.ac.kr; 5 Hanyang Institute of Bioscience and Biotechnology, Hanyang University, #222-1 Wangsimni-ro, Seongdong-gu, Seoul 04763, Republic of Korea

**Keywords:** curcumin, diabetic retinopathy, inflammasome

## Abstract

**Background**: Diabetic retinopathy (DR) is a leading cause of vision loss, with oxidative stress and inflammation serving as critical drivers of its onset and progression. Curcumin, a natural polyphenol, is known for its potent anti-inflammatory and antioxidant properties. Glial fibrillary acidic protein (GFAP) is commonly used as an indicator of retinal macroglial reactivity, whereas the NLRP3 inflammasome is an important mediator of inflammatory signaling in DR. We evaluated retinal vascular abnormalities and retinal GFAP immunoreactivity and examined whether these findings were accompanied by changes in retinal NLRP3 and IL-1β protein abundance and in the levels of cleaved caspase-1 and cleaved GSDMD. **Methods**: Diabetes was induced in 7-week-old rats via a single intraperitoneal injection of streptozotocin. Following the confirmation of hyperglycemia, curcumin was administered orally. To evaluate the effects, we performed trypsin digestion, haematoxylin and eosin (H&E) staining, fluorescein isothiocyanate (FITC)–dextran permeability assays, immunofluorescence staining, and Western blotting on the harvested retinas. **Results**: The DM group showed increased acellular capillary formation, vascular leakage, retinal GFAP immunoreactivity, NLRP3 and IL-1β protein abundance, and levels of cleaved caspase-1 and cleaved GSDMD. Compared with the untreated DM group, the DM + curcumin group showed lower values for all of these outcomes. **Conclusions**: In diabetic rats, oral administration of a curcumin-containing formulation was associated with fewer retinal vascular abnormalities and lower GFAP immunoreactivity, accompanied by parallel differences in inflammasome-related proteins. These findings support the potential of this formulation as an adjunctive therapeutic approach for early diabetic retinopathy.

## 1. Introduction

Diabetic retinopathy (DR) is a complication of diabetes and major cause of blindness. Owing to the persistence of diabetes-induced hyperglycemia, damage occurs in the retinal microvasculature. Consequently, ischemic damage occurs in the retina, causing DR. As DR progresses, neovascularization occurs causing blindness [1].

Curcumin (1,7-bis(4-hydroxy-3-methoxyphenyl)-1,6-heptadiene-3,5-dione) is a natural phenolic compound that is primarily found in Indian turmeric and is associated with powerful anti-inflammatory and antioxidant properties. According to traditional medicine, curcumin plays an anti-inflammatory role in several diseases [2,3]. In diabetes, curcumin treatment ameliorates complications, such as diabetic cardiomyopathy [4], diabetic encephalopathy [5], diabetic nephropathy [6], and DR [7,8]. Previous studies of DR have reported that curcumin enhances antioxidant defenses, including superoxide dismutase and catalase, reduces the proinflammatory cytokine TNF-α, and inhibits VEGF expression [7,8].

Hyperglycemia promotes oxidative stress, inflammatory signaling, vascular abnormalities, and glial responses in the retina [9]. Assembly of the NLRP3 inflammasome promotes the cleavage and activation of procaspase-1, and activated caspase-1 subsequently mediates IL-1β maturation and GSDMD cleavage [10,11]. The resulting pore-forming GSDMD N-terminal fragment is a key executor of pyroptosis-associated inflammatory signaling [11,12]. Given the antioxidant and anti-inflammatory properties of curcumin, we evaluated retinal vascular abnormalities and retinal GFAP immunoreactivity and examined the accompanying changes in retinal NLRP3 and IL-1β protein abundance and in the levels of cleaved caspase-1 and cleaved GSDMD.

Curcumin has been reported to ameliorate white matter injury after ischemic stroke by suppressing NF-κB/NLRP3-associated microglial pyroptosis [13]. Curcumin has also attenuated kidney inflammation in a hyperuricemia model in association with inhibition of NLRP3 signaling [14]. However, these findings from other disease models do not establish that the retinal protective effects of curcumin are NLRP3-dependent. Accordingly, the present study was designed to assess concurrent retinal structural, glial, and inflammasome-related protein changes following oral curcumin administration, rather than to establish NLRP3 pathway dependence.

## 2. Methods

### 2.1. Animals

All animal experiments were reviewed and approved by the Institutional Animal Care and Use Committee (IACUC) of the College of Medicine, Hanyang University (IACUC Approval No. HY-IACUC-2021-0251A). Seven-week-old Wistar male rats were purchased from Orient Bio (Seongnam, Republic of Korea). Rats were housed at 22 °C and 60% humidity under a 12 h light–dark cycle and fed food and water ad libitum. Animals were given a 1-week adaptation period before being used in the experiment.

### 2.2. Introduction of Experimental Diabetes in Rats and Curcumin Administration

Diabetes was induced in rats via a single intraperitoneal injection of streptozotocin (STZ) (65 mg/kg, Sigma, St. Louis, MO, USA) dissolved in 0.1 M citrate buffer (pH 4.5); the normal group was intraperitoneally administered with 0.1 M citrate buffer as the control. At 72 h after STZ injection, the fasting blood glucose (BG) level of rats was measured, and a BG level of ≥300 mg/dL indicated successful development of the diabetes model. The fasting BG level of rats was measured once per week for 8 weeks to confirm disease persistence. Rats were fasted for 12 h before measurement of their BG levels. Diabetic rats were randomly divided into the diabetes mellitus (DM) and DM + curcumin groups.

Rats in the DM + curcumin group received curcumin orally at 200 mg/kg/day for 4 weeks, beginning 4 weeks after STZ injection. This dose was selected based on previous studies reporting retinal protective effects of orally administered curcumin at 200 mg/kg/day in STZ-diabetic rats [15,16]. Curcumin was freshly suspended in 0.5% hypromellose (Sigma) before administration. The untreated DM group received neither curcumin nor hypromellose. All rats were euthanized 8 weeks after STZ injection.

### 2.3. Trypsin Digestion and Haematoxylin and Eosin (H&E) Staining

Enucleated eyeballs were fixed in 4% paraformaldehyde (PFA) for one day. Retinas were then dissected and washed four times with filtered distilled water (DW) for 30 min. After overnight washing at room temperature (21–23 °C), retinas were treated with 3% trypsin (Gibco, Waltham, MA, USA) in 0.1 M Tris-Cl buffer (pH 7.5) for 1 h and incubated at 37 °C. Following trypsin removal, retinas were washed with DW using a pipette. Retinal vessels were transferred onto microslides and stained with 0.1% Gill’s haematoxylin (Sigma) for 3 min, washed with DW for 5 min, and immersed in 0.5% eosin (Sigma) 12 times. The retinas were then submerged two times in DW to remove eosin, sequentially immersed in 50, 70, 95, and 100% ethanol 10 times, and finally cleared in xylene for at least 10 s. Fifteen micrographs were captured per subject, and the number of acellular capillaries was counted and averaged. Images were acquired using a microscope (BX53F, Olympus, Tokyo, Japan).

### 2.4. Fluorescein Isothiocyanate (FITC)–Dextran Permeability Assay

FITC–dextran (50 mg/kg, Sigma) was intravenously administered to rats 1 h before euthanization. Eyeballs were enucleated from rats euthanized via CO_2_ asphyxiation and then stored in 4% PFA at 4 °C for 24 h. Retinas separated from the eyeballs were flattened and mounted using mounting medium (Fisher Chemical, Hampton, VA, USA) and then photographed using a confocal fluorescence microscope (K1-fluo, Nanoscope Systems Inc., Daejeon, Korea; DMIRB, Leica, Wetzlar, Germany). Three non-overlapping micrographs were acquired from spatially separated regions of each retinal flat mount using identical magnification, laser power, detector gain, and exposure settings across all groups. Fields were selected without reference to the presence or intensity of focal leakage. Mean FITC fluorescence intensity was quantified across the full image field using ImageJ 1.53k. The three field values were averaged to generate one value per animal.

### 2.5. Immunofluorescence Staining

After euthanization, rats were perfused with 4% PFA. Eyeballs were then excised, treated with 4% PFA, and incubated overnight in 30% sucrose solution. Eyeballs were then embedded in optimal cutting temperature compound (Sakura Finetek, Torrance, CA, USA) and then cryosectioned to a 15 μm thickness at −20 °C. Tissues were washed with phosphate-buffered saline, fixed in 4% PFA for 15 min, and blocked with 5% bovine serum albumin (BSA; GenDEPOT, Katy, TX, USA) for 1 h at room temperature. Tissues were then incubated with glial fibrillary acidic protein (GFAP) antibody (ab7260, Abcam, Cambridge, UK) (1:1000) diluted in 5% BSA overnight at 4 °C, and then diluted in 5% BSA for 1 h at room temperature with Alexa 488 anti-rabbit secondary antibody (A11008, Invitrogen, Waltham, MA, USA). Samples were mounted using mounting medium with 4′,6-diamidino-2-phenylindole (DAPI) (P36931, Invitrogen) and hardened at 4 °C for 5 h. Retinas were photographed using a fluorescence microscope (Eclipse Ti, Nikon, Tokyo, Japan). Three micrographs were captured per animal. In each image, the region of interest encompassed the full retinal thickness, and the mean GFAP fluorescence intensity within the retinal tissue was quantified using ImageJ. The values obtained from the three images were averaged to generate one value per animal.

### 2.6. Western Blotting

Rats were euthanized via CO_2_ asphyxiation, and retinas were removed from enucleated eyes, stored at −70 °C, and then lysed with RIPA buffer (Sigma), protease inhibitor cocktail (Thermo Fisher, Waltham, MA, USA), and 0.5 M ethylene–diamine–tetraacetic acid (EDTA) solution (Thermo Fisher) in a homogeniser. Retina samples were sonicated using 10 cycles of 10 s on/10 s off at 100 W and centrifuged at 15,000 rpm for 10 min at 4 °C. The bicinchoninic acid assay was performed to quantify the level of proteins in the supernatant. Sodium dodecyl sulphate–polyacrylamide gel electrophoresis was performed at 80 V for 3 h to separate the proteins. Electrophoresed proteins were transferred onto a 0.2 μm polyvinylidene fluoride membrane at 100 V for 90 min. Membranes were blocked with 5% skim milk for 1 h, incubated overnight at 4 °C with primary antibodies diluted in 5% BSA, and then incubated with a horseradish peroxidase-conjugated anti-rabbit secondary antibody (#711-035-152, Jackson ImmunoResearch Laboratories Inc., West Grove, PA, USA) diluted in 5% BSA for 2 h at room temperature. The primary antibodies used in this study were as follows: rabbit anti-NLRP3 (1:500, #NBP2-12446, Novus Biologicals, Littleton, CO, USA), rabbit anti-IL-1β (1:500, #ab9722, Abcam, Cambridge, UK), rabbit anti-caspase-1 (1:500, #NBP1-45433, NOVUS Biologicals), rabbit anti-GSDMD (1:500, #ab219800, Abcam), and rabbit anti-β-actin (1:2000, #4967S, Cell Signaling Technology, Beverly, MA, USA). Protein bands were visualized using an ImageQuant LAS 4000 system (GE Healthcare Bio-Sciences AB, Uppsala, Sweden). The band intensities of NLRP3, IL-1β, cleaved caspase-1, and the GSDMD N-terminal fragment (GSDMD-N) were quantified and normalized to β-actin. After GSDMD detection and image acquisition, the membrane was stripped using 0.1M Glycine (Sigma) for 10 min at room temperature, washed, blocked again, and incubated with the β-actin antibody. The original uncropped Western blot images corresponding to these analyses are provided in Supplementary Figure S1.

### 2.7. Statistical Analysis

Data are presented as the mean ± standard deviation. For image-based analyses, measurements obtained from multiple fields of the same retina were averaged to generate one value per animal before statistical testing. Statistical analyses were performed using Prism 8 (GraphPad Software Inc., San Diego, CA, USA). Multiple comparisons were performed using one-way or two-way analysis of variance, followed by Tukey’s post hoc test, as appropriate. Statistical significance was set at *p* < 0.05. For clarity, group means ± standard deviations are shown in the main longitudinal plots.

## 3. Results

### 3.1. BG and Body Weight of Diabetic Rats

**Figure 1:**
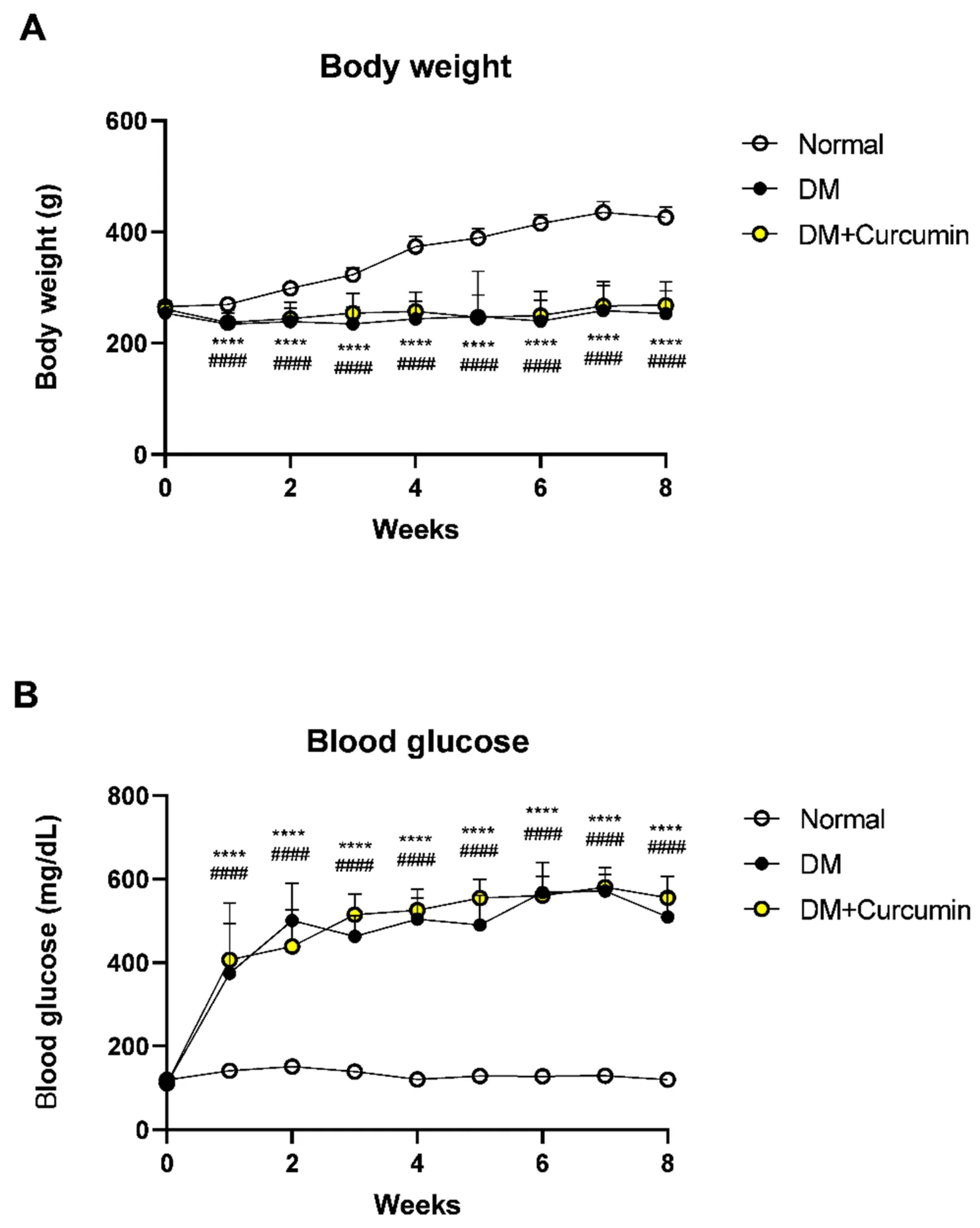
Body weight and blood glucose levels of streptozotocin-induced diabetic rats. The body weight of the normal group increased over time while that of the streptozotocin (STZ)-induced diabetes mellitus (DM) group and DM + curcumin group decreased immediately after STZ injection; a trend in increasing body weight was not found for rats administered STZ (**A**). Compared with those in the normal group, blood glucose levels significantly increased in the DM and DM + curcumin groups, although no difference was found between these groups (**B**). Results are presented as means ± standard deviations and analyzed using two-way analysis of variance. Normal; n = 14, DM; n = 14, DM + curcumin; n = 12, **** *p* <0.0001, #### *p* <0.0001.

The fasting BG level and body weight (BW) of diabetic rats were measured once per week after 12 h of fasting. The BW of rats in the normal group increased over time while that of rats in the diabetic group decreased; the BW in the diabetic group was significantly lower than that in the normal group, which steadily increased (*p* < 0.0001). After 4 weeks, diabetic rats were randomly divided into two groups: DM and DM + curcumin groups. No significant difference in BW was found between these groups. The BG level of the normal group was 131.93 ± 10.81 mg/dL and did not significantly change for 8 weeks. The BG level of the DM group significantly increased compared with that of the normal group (*p* < 0.0001). No significant difference in the BG level was found between the DM and DM + curcumin groups (Figure 1). The corresponding individual animal-level body-weight and blood-glucose measurements at each time point are provided in Supplementary Figure S2.

### 3.2. Trypsin Digestion

**Figure 2:**
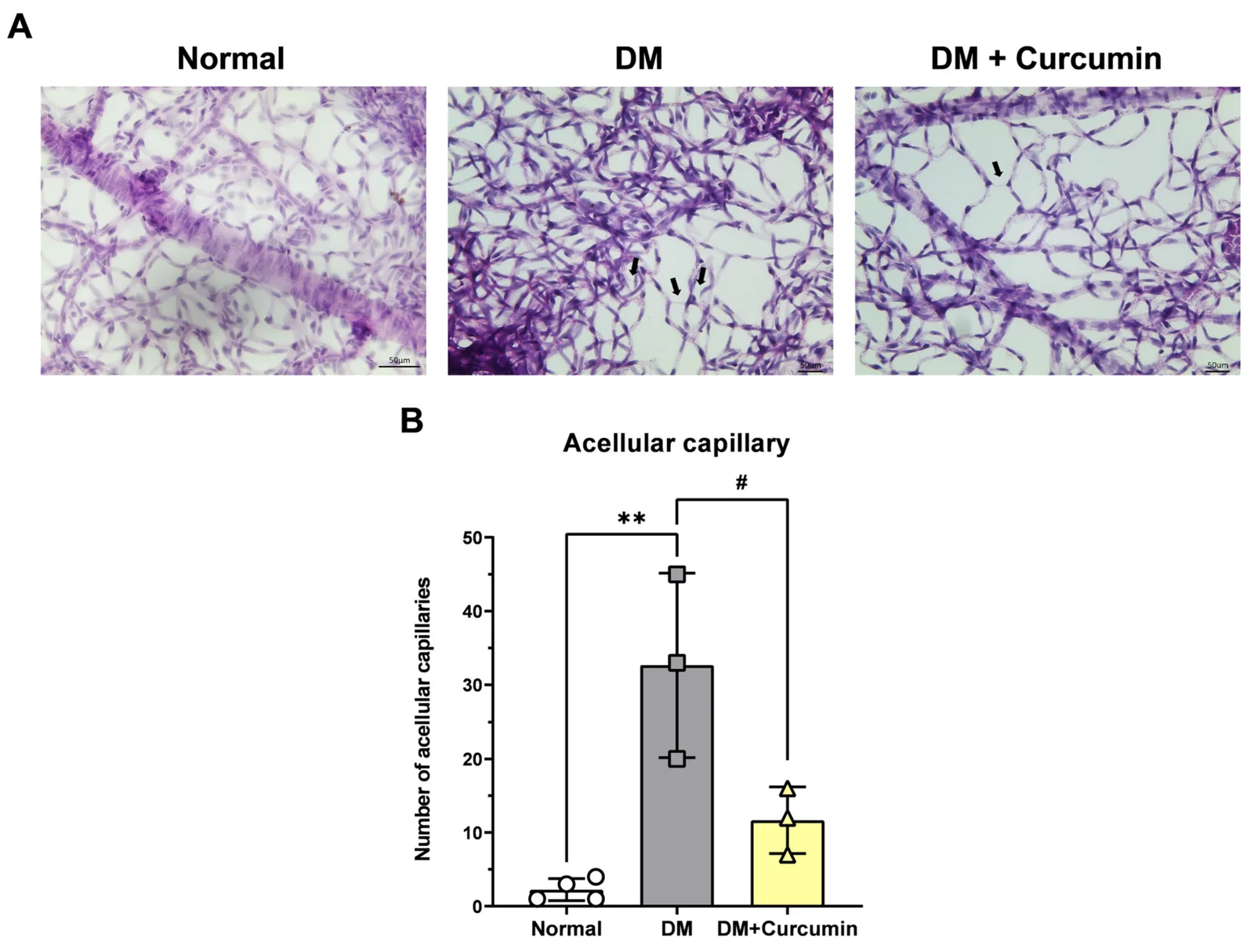
Retinal acellular capillary counts in the experimental groups. Black arrows indicate acellular capillaries (**A**). A significantly higher number of acellular capillaries was found in retinas of rats in the diabetes mellitus (DM) group than in retinas of rats in the normal group while a significantly lower number of acellular capillaries was identified in the retina of rats in the DM + curcumin group than in the retinas of rats in the DM group (**B**). Each point represents one animal. Results are presented as means ± standard deviations and analyzed using one-way analysis of variance. Normal; n = 4, DM; n = 3, DM + curcumin; n = 3, ** *p* <0.01, # *p* <0.05, scale bar: 50 µm.

The trypsin digestion confirmed the existence of retinal acellular capillaries, a pathological change associated with DR. When only the retinal vascular system was retained, the presence of acellular capillaries could be visualised via H&E staining. Fifteen microscopic images were captured per subject, and the number of acellular capillaries was counted and averaged. The DM group had significantly more acellular capillaries than the normal group (*p* < 0.01). The DM + curcumin group had significantly fewer acellular capillaries than the untreated DM group (*p* < 0.05; Figure 2).

### 3.3. FITC Permeability Assay

**Figure 3:**
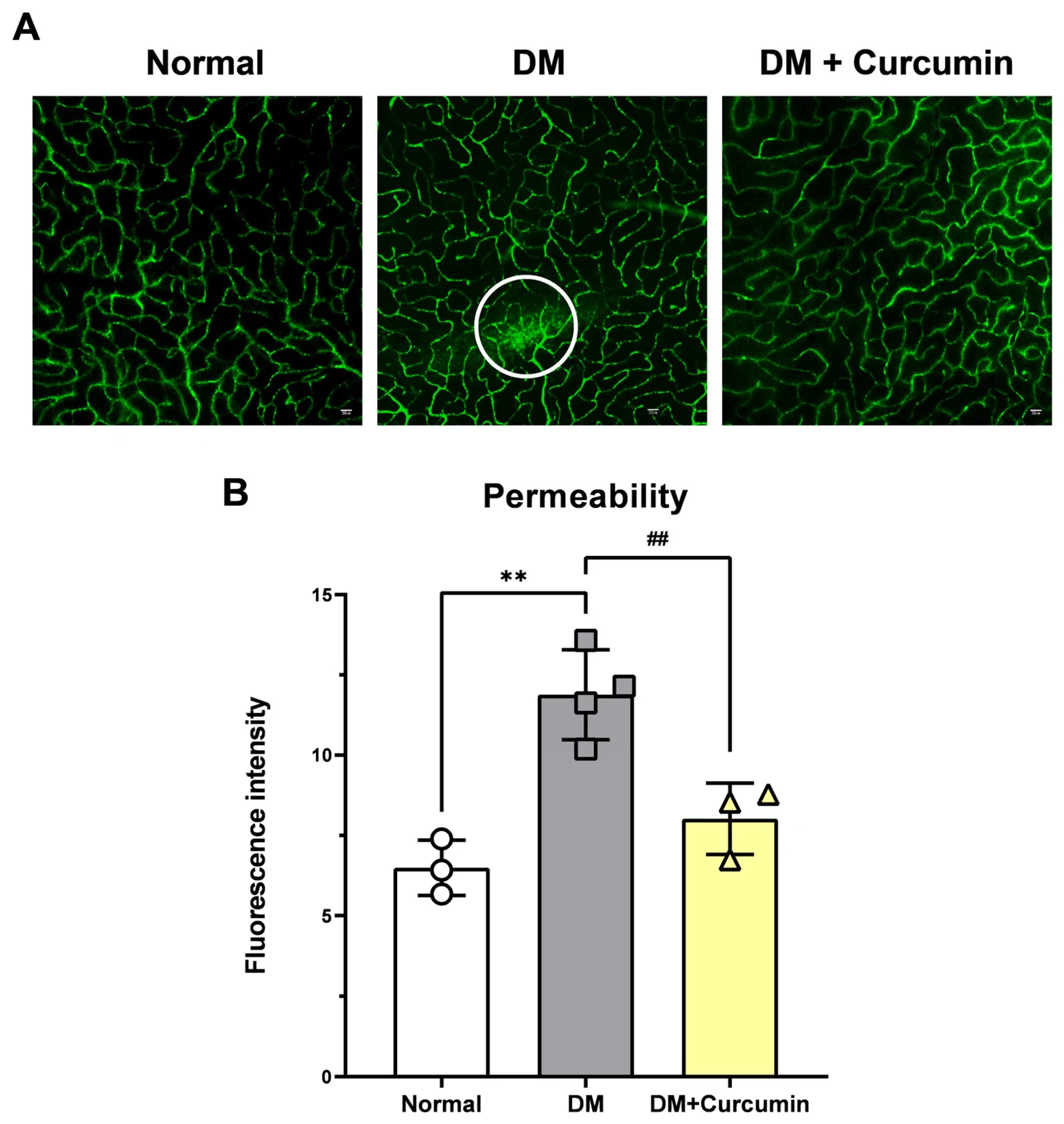
Retinal vascular permeability in the experimental groups. The white circle identifies a representative leakage focus for visual orientation (**A**) and was not used as the quantitative ROI boundary. Vascular leakage was significantly greater in the retinas of rats in the diabetes mellitus (DM) group than that in the retinas of rats in the normal group, whereas vascular leakage was significantly lower in the retinas of rats in the DM + curcumin group than that in the DM group (**B**). Each point represents one animal. Results are presented as means ± standard deviations and analyzed using one-way analysis of variance. Normal; n = 3, DM; n = 4, DM + curcumin; n = 3, ** *p* <0.01, ## *p* <0.01, scale bar: 30 µm.

A vascular permeability assay was performed to detect microvascular leakage, a pathological change of DR, in the retina. The mean fluorescence intensity was significantly higher in the DM group than in the normal group (*p* < 0.01), indicating greater microvascular leakage in the DM group. The mean fluorescence intensity was significantly lower in the DM + curcumin group than in the untreated DM group (*p* < 0.01; Figure 3).

### 3.4. Immunofluorescence Staining

**Figure 4:**
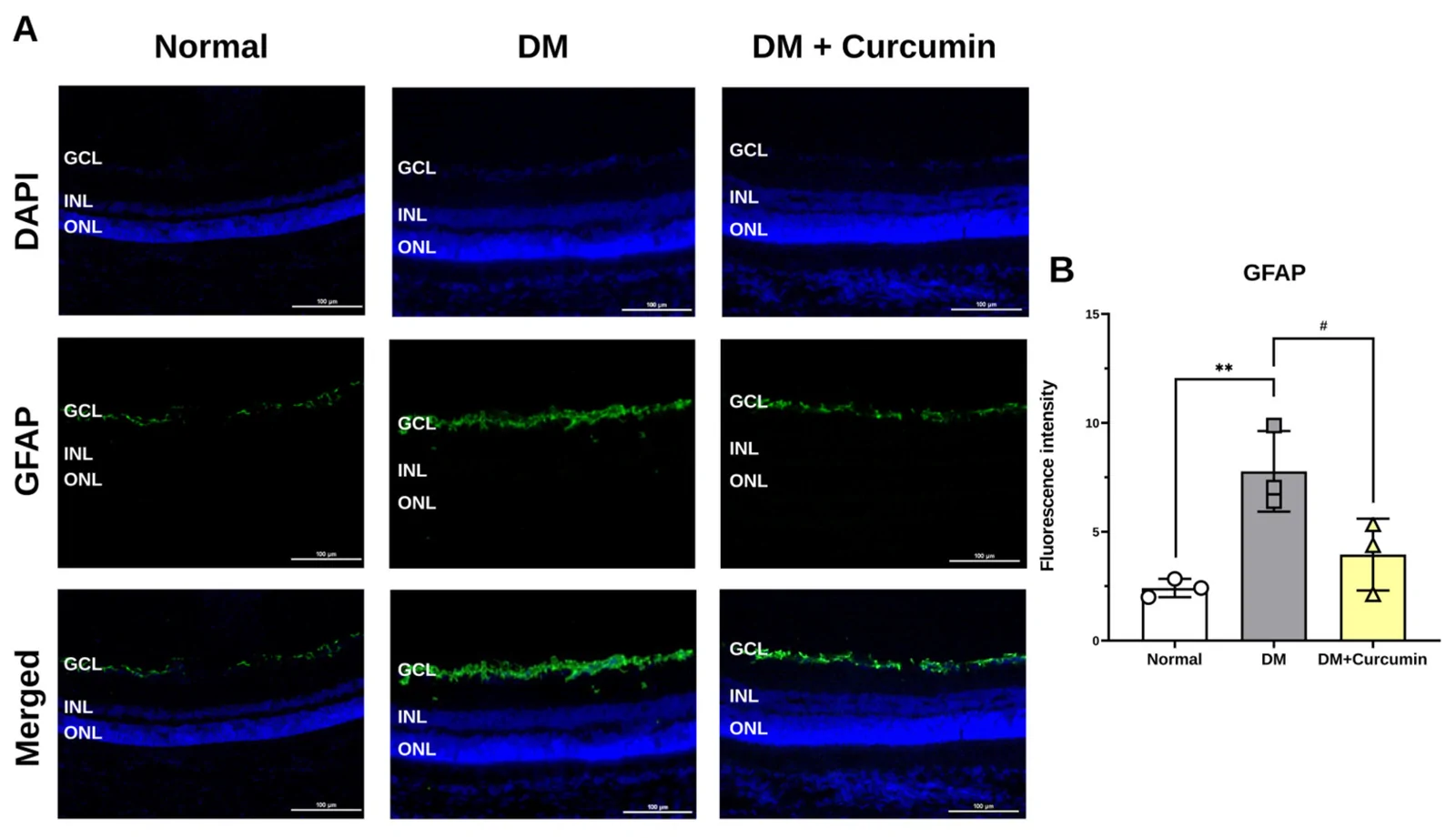
Retinal GFAP immunoreactivity in the experimental groups. (**A**) Representative retinal sections stained for glial fibrillary acidic protein (GFAP) are shown. The mean GFAP fluorescence intensity was quantified within a full retinal-thickness region of interest. (**B**) GFAP immunoreactivity was significantly higher in the diabetes mellitus (DM) group than in the normal group and was significantly lower in the DM + curcumin group than in the DM group. Each point represents one animal. The measured signal represents retinal GFAP immunoreactivity and does not identify a specific glial cell population. GFAP immunoreactivity is shown in green, and nuclei stained with DAPI are shown in blue. Data are presented as the mean ± standard deviation and were analyzed using one-way analysis of variance followed by Tukey’s post hoc test. Normal, n = 3; DM, n = 3; DM + curcumin, n = 4. ** *p* < 0.01 versus the normal group; # *p* < 0.05 versus the DM group.

GFAP immunofluorescence was quantified across the full retinal-thickness region of interest. The mean retinal GFAP fluorescence intensity was significantly higher in the DM group than in the normal group (*p* < 0.01) and was significantly lower in the DM + curcumin group than in the untreated DM group (*p* < 0.05; Figure 4).

### 3.5. Western Blotting

**Figure 5:**
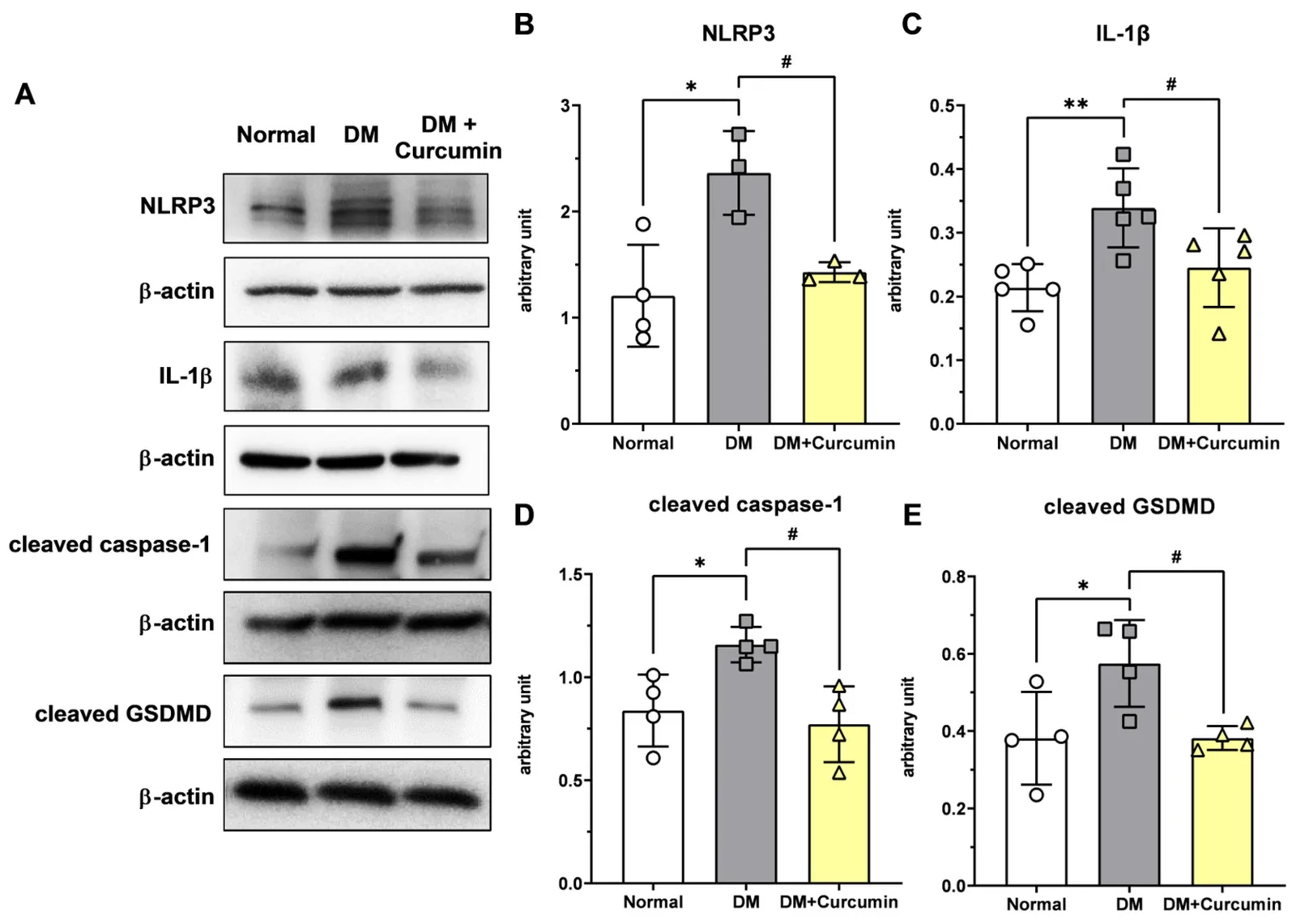
(**A**) Retinal NLRP3, IL-1β, cleaved caspase-1, and cleaved GSDMD levels in the experimental groups. Retinal (**B**) NLRP3 and (**C**) IL-1β protein abundance and the levels of (**D**) cleaved caspase-1 and (**E**) GSDMD-N were higher in the diabetes mellitus (DM) group than in the normal group and were lower in the DM + curcumin group than in the untreated DM group. Target protein levels were normalized to β-actin, and each point represents one animal. Data are presented as the mean ± standard deviation. Statistical comparisons were performed using one-way analysis of variance followed by Tukey’s post hoc test. Sample sizes were as follows: NLRP3, n = 4, 3, and 3; IL-1β, n = 5, 4, and 4; cleaved caspase-1, n = 4, 4, and 4; and cleaved GSDMD, n = 4, 3, and 5 for the normal, DM, and DM + curcumin groups, respectively. * *p* < 0.05 and ** *p* < 0.01 versus the normal group; # *p* < 0.05 versus the DM group.

Retinal NLRP3 and IL-1β protein abundance and the levels of cleaved caspase-1 and GSDMD-N were assessed by Western blotting. Compared with the normal group, the DM group showed significantly higher levels of all four measurements. All four measurements were significantly lower in the DM + curcumin group than in the untreated DM group (Figure 5). The original uncropped Western blot images corresponding to these analyses are provided in Supplementary Figure S1.

## 4. Discussion

In this study, retinal vascular abnormalities, retinal GFAP immunoreactivity, and inflammasome-related protein levels were compared among the experimental groups. Compared with the normal group, the DM group showed more acellular capillaries, greater retinal vascular leakage, higher retinal GFAP immunoreactivity, and higher retinal levels of NLRP3, IL-1β, cleaved caspase-1, and GSDMD-N. Compared with the untreated DM group, the DM + curcumin group showed lower values for each of these outcomes.

DR is caused by high blood sugar levels, which induce oxidative stress, causing retinal cell death and activating glial cells. Activated glial cells activate the NLRP3 inflammasome and secrete inflammatory cytokines. Hyperglycemia-induced oxidative stress and glial responses can promote inflammasome-related signaling in the diabetic retina [9]. In canonical NLRP3 signaling, assembly of the NLRP3–ASC–procaspase-1 complex promotes caspase-1 cleavage. Activated caspase-1 can subsequently process pro-IL-1β and GSDMD, and the pore-forming GSDMD N-terminal fragment can contribute to pyroptotic cell death under appropriate cellular conditions [11,17,18,19]. These established pathway relationships provide a biological context but do not demonstrate that the complete pathway occurred in the retinas examined in the present study.

In the present study, NLRP3 and IL-1β protein abundance and the levels of cleaved caspase-1 and GSDMD-N were higher in the DM group and lower in the DM + curcumin group. Because no NLRP3-selective pharmacological or genetic manipulation was performed, these parallel molecular changes do not establish that NLRP3 is required for, or causally mediates, the observed retinal phenotype. The findings are therefore interpreted as treatment-associated changes in inflammasome-related proteins rather than evidence of an NLRP3-dependent protective mechanism.

GFAP immunoreactivity is commonly used to assess retinal macroglial responses, and diabetes can alter GFAP expression in both retinal astrocytes and Müller cells [20,21]. Previous work has also reported reduced retinal GFAP expression after curcumin treatment in diabetic rats [22]. In the present study, the mean GFAP fluorescence intensity across the full retinal-thickness region of interest was higher in the DM group than in the normal group and lower in the DM + curcumin group than in the untreated DM group. Because cell-specific co-labeling was not performed, the observed signal cannot be assigned to a specific glial population. We therefore describe this outcome as retinal GFAP immunoreactivity rather than gliosis or Müller cell activation.

The DM + curcumin group showed fewer acellular capillaries and lower retinal vascular leakage than the untreated DM group. These between-group differences may reflect changes in multiple components of the retinal neurovascular unit. However, the present measurements do not identify the specific retinal cell populations involved.

Because curcumin treatment began 4 weeks after induction of diabetes and retinal outcomes were assessed at 8 weeks, the intervention targeted established early retinal abnormalities rather than preventing the onset of hyperglycemia. Short-duration STZ models at this stage demonstrate early retinal glial responses and blood–retinal barrier abnormalities [21,23,24]. In the present study, retinal GFAP immunoreactivity, vascular leakage, and acellular capillary formation were observed, whereas retinal neovascularization was neither assessed nor demonstrated. Therefore, this model is best regarded as representing early, pre-neovascular DR-like pathology, broadly corresponding to early non-proliferative diabetic retinopathy at the level of neuroinflammatory and vascular-barrier abnormalities. This comparison is pathophysiological rather than a direct clinical stage assignment because human NPDR is graded using funduscopic features that are not fully reproduced in short-duration STZ models.

Blood glucose and body weight did not differ significantly between the DM and DM + curcumin groups. Thus, the retinal differences between these groups occurred despite persistent hyperglycemia and were not accompanied by a measurable correction of systemic blood glucose. A similar dissociation was reported by Kowluru and Kanwar, who observed reduced retinal oxidative stress and inflammatory mediators after curcumin administration without changes in blood glucose or body weight [25]. Curcumin has also been reported to reduce retinal vascular leakage and the expression of VEGF, iNOS, and ICAM-1 in STZ-diabetic rats and high-glucose-exposed Müller cells [26]. These previous findings provide biological context for retinal modulation downstream of hyperglycemia. However, because systemic inflammatory markers, retinal curcumin concentrations, and cell-specific targets were not assessed, the present data do not establish a direct or retina-restricted mechanism.

VEGF is a major mediator of retinal vascular permeability and, in advanced disease, pathological neovascularization. Curcumin has been reported to reduce retinal VEGF expression in STZ-diabetic rats and to suppress VEGF, iNOS, and ICAM-1 through CaMKII/NF-κB signaling in diabetic rats and high-glucose-exposed Müller cells [26]. NLRP3/IL-1β-associated inflammation and VEGF signaling may therefore converge on blood–retinal barrier dysfunction rather than acting as mutually exclusive pathways. Consistent with this interpretation, NLRP3-related inflammatory markers and pro-angiogenic markers coexist in advanced DR models, whereas suppression of TXNIP/NLRP3 signaling reduces retinal inflammatory mediators and vascular permeability [27,28]. However, VEGF was not measured in the present study, and no conclusion can be drawn as to whether the lower vascular leakage observed in the DM + curcumin group was VEGF-dependent.

The oral dose of 200 mg/kg/day was selected because previous studies in STZ-diabetic rats reported retinal structural, antioxidant, anti-apoptotic, and VEGF-related effects at this dose, including a study using a 4-week treatment period [15,16]. Although unformulated curcumin has limited systemic bioavailability because of poor absorption and rapid metabolism, curcumin has been detected in ocular tissues following oral administration in rats, and retinal delivery has been shown to depend strongly on formulation [29,30]. Because retinal curcumin concentrations were not measured in the present study, the actual retinal exposure and blood–retinal barrier penetration of curcumin cannot be determined from our data.

Body weight did not differ significantly between the DM and DM + curcumin groups. However, the present experiment was not designed as a formal toxicology study, and clinical chemistry, hematology, and non-retinal organ histology were not assessed. Although hypromellose is a poorly absorbed cellulose derivative with a generally low oral toxicity profile [31,32], this general safety information does not exclude a retinal-specific effect of the vehicle. Moreover, the absence of differences in body weight or blood glucose between the DM and DM + curcumin groups does not formally exclude such an effect. Because the untreated DM group received neither curcumin nor hypromellose, the comparison between the two diabetic groups does not isolate the effect of curcumin from a potential contribution of the vehicle. The observed retinal differences therefore cannot be attributed exclusively to curcumin.

Current treatments for DR include the intravitreal injection of anti-VEGF drugs (antiangiogenic therapy), intravitreal injection of steroids (anti-inflammatory therapy), and laser treatment [33]. VEGF plays a role in the generation of new blood vessels within the body. VEGF overexpression in the eye can induce the growth of abnormal blood vessels, whereas anti-VEGF treatment slows the growth and damage of blood vessels in the eye [33]. However, the response to intravitreal anti-VEGF treatment varies among patients and is difficult to predict, and a proportion of patients do not respond to treatment [34]. As potent anti-inflammatory agents, corticosteroids target various mediators involved in the pathogenesis of diabetic macular oedema, including VEGF, TNF-α, and chemokines [33]. However, owing to their high incidence of side effects, corticosteroids are generally considered a second-line agent for patients who do not respond sufficiently to other treatments [33]. Finally, laser treatment can cause permanent damage to retinal cells, causing side effects, such as mild loss of central vision and reduced night vision [35]. Once DR occurs, normal conditions cannot be restored, although DR progression can be slowed if BG levels and accompanying diseases are well-controlled. Therefore, the goal of DR treatment involves the careful and regular monitoring of the progression of the condition, administration of treatment at the appropriate time to slow progression, and preservation of vision for as long as possible. These observations support further controlled evaluation of curcumin-containing formulations as potential adjunctive approaches for retinal inflammation.

The present study has several limitations. Although cleaved caspase-1 and GSDMD-N were assessed, no NLRP3-selective pharmacological or genetic manipulation was performed; therefore, whether NLRP3 is required for the retinal differences observed between the untreated DM and DM + curcumin groups remains unconfirmed. Retinal ROS, TXNIP, and IL-18 were not measured, preventing an assessment of the upstream trigger and the full extent of inflammatory cytokine maturation. GFAP was quantified across the full retinal-thickness region of interest without cell-specific co-labeling, and the relative contributions of Müller cells and astrocytes remain unclear. Likewise, whole-retina lysates do not identify the retinal cell population directly affected by curcumin. Because the untreated DM group received neither curcumin nor hypromellose, and a separate DM + vehicle group was not included, the comparison between the DM and DM + curcumin groups does not isolate the effect of curcumin. A retinal contribution of hypromellose cannot be excluded, and the observed differences cannot be attributed exclusively to curcumin. Future studies using NLRP3-selective pathway manipulation, cell-specific models, and retinal pharmacokinetic measurements are required.

Overall, compared with untreated diabetic rats, rats in the DM + curcumin group showed fewer retinal vascular abnormalities, lower GFAP immunoreactivity, and lower levels of NLRP3, IL-1β, cleaved caspase-1, and GSDMD-N. These concurrent retinal and molecular differences do not establish that NLRP3 causally mediates the observed retinal phenotype.

## 5. Conclusions

Compared with untreated diabetic rats, rats in the DM + curcumin group showed fewer retinal acellular capillaries, lower vascular leakage and GFAP immunoreactivity, and lower retinal levels of NLRP3, IL-1β, cleaved caspase-1, and GSDMD-N. These molecular findings do not establish NLRP3-dependent causality, mature IL-1β generation, or direct pyroptotic cell death. In addition, because a separate DM + hypromellose group was not included, the observed retinal differences cannot be attributed exclusively to curcumin. Further studies incorporating NLRP3-specific pathway manipulation, direct assessment of pyroptotic cell death, and an appropriate diabetic vehicle-control group are required.

## Data Availability

The original contributions presented in this study are included in the article. Further inquiries can be directed to the corresponding authors.

## Supplementary Materials

The following supporting information can be downloaded at: https://www.mdpi.com/article/10.3390/life16091427/s1, Figure S1. Original uncropped Western blot images for NLRP3, IL-1β, cleaved caspase-1, cleaved GSDMD, and the corresponding loading controls. Molecular-weight markers and lane assignments are indicated. Figure S2. Individual longitudinal body-weight and blood-glucose measurements for each animal.
